# Role of Myeloid Cell-Specific Adenylyl Cyclase Type 7 in Lipopolysaccharide- and Alcohol-Induced Immune Responses

**DOI:** 10.3390/ijms252312831

**Published:** 2024-11-28

**Authors:** Yawen Hu, Sonika Patial, Yogesh Saini, Masami Yoshimura

**Affiliations:** Department of Comparative Biomedical Sciences, School of Veterinary Medicine, Louisiana State University, Baton Rouge, LA 70803, USA; yhu2@lsuhsc.edu (Y.H.); sonika.patial@nih.gov (S.P.); ysaini@ncsu.edu (Y.S.)

**Keywords:** adenylyl cyclase type 7, LysMcre, conditional knockout mice, alcohol, lipopolysaccharide, survival, cytokine expression

## Abstract

Clinical and experimental evidence indicates that alcohol use causes various abnormalities in the immune system and compromises immune functions. However, the mechanistic understanding of ethanol’s effects on the immune system remains limited. Cyclic AMP (cAMP) regulates multiple processes, including immune responses. Earlier research indicated that type 7 adenylyl cyclase (AC7) regulates the immune system and is highly responsive to ethanol. Therefore, we hypothesized that AC7 is a central player in regulating the effects of alcohol on innate immune responses. To test this hypothesis, we utilized a myeloid lineage-specific AC7 KO mouse model and compared the effects of acute and chronic ethanol treatment on their innate immune responses induced by systemic lipopolysaccharide (LPS) challenge. Our results demonstrate that AC7 KO mice had significantly lower survival rates under LPS challenge. Chronic ethanol consumption rescued AC7 KO mice from LPS-induced death. AC7 KO and ethanol, acute and chronic, affected several measurements of cytokine mRNA expressions, including IL-1β, TNFα, IL-6, and IL-10 in the lung and liver. In a few cases, statistical analysis indicated that these two factors interacted, suggesting that AC7 played some role in ethanol’s effect on cytokine expression. Thus, this study demonstrated AC7’s role in ethanol’s effect on the innate immune response.

## 1. Introduction

Alcohol consumption compromises the function of various components of the immune defense system by modulating innate and adaptive immunity. In general, acute ingestion of alcohol is immunosuppressive [1]. Depending on the duration and quantity of alcohol usage, a wide range of changes in the immune system can occur due to chronic alcohol consumption. As reviewed by many researchers [2,3,4], alcohol use increases the incidence of infections caused by bacteria and viruses and can negatively affect recovery from infections and traumatic injuries. Lung diseases present particularly interesting examples associated with alcohol intoxication [5,6,7,8,9,10]. People who consume alcohol are highly prone to lung infections, including bacterial pneumonia and tuberculosis. In fact, alcohol abuse is a major risk factor for the development of pulmonary diseases. Bacterial pneumonia and other lung infections associated with alcohol abuse are, in general, more severe and result in higher morbidity and mortality. However, the mechanisms responsible for ethanol’s effects on the immune system are not fully understood.

Cyclic AMP (cAMP) is a master regulator of immune cell function, exerting inhibitory effects on innate and adaptive immune responses. In the lung, cAMP suppresses inflammatory mediator generation, microbicidal activity, phagocytosis, and neutrophil recruitment [11]. In mammals, there are ten isoforms of adenylyl cyclase (AC). Among these, types 1–9 are G protein-regulated transmembrane ACs, while type 10 is a soluble AC regulated by calcium and bicarbonate [12]. Type 7 AC (AC7) is somewhat unique in that it is activated by four different classes of heterotrimeric G proteins, namely, G_s_, G_i/o_, G_q_, and G_12/13_ [12,13]. G_13_ signaling requires AC7 to enhance intracellular cAMP production in the immune system [14]. In previous studies, we have established that AC7 is the most ethanol-responsive AC isoform [15,16,17,18,19]. In the presence of G_s_ activation, ethanol at physiologically relevant concentrations (less than 50 mM) can stimulate the activity of AC7 [15,20], which results in an increase in protein kinase A activity [20] and an increase in cAMP-regulated gene expression [18]. AC7 is highly expressed in immune cells, including macrophages, neutrophils, T cells, and B cells [14,21,22]. Because of AC7’s high responsiveness to ethanol and its high expression in immune cells, we hypothesize that AC7 plays a vital role as a mediator of ethanol’s effects on immune responses.

We and others generated whole-body conventional AC7 knockout (KO) mice and found that homozygous AC7 knockout mice exhibit a perinatal or juvenile lethal phenotype [21,23], suggesting that AC7 is essential for the survival of mice and that these mice are not suitable animals for research. Conditional AC7 knockout mice specific to myeloid lineage cells were developed by breeding mice bearing loxP-flanked AC7 alleles with mice that express LysMcre transgene [14]. The LysMcre transgene used in this project is reported to be expressed in the cells of the myeloid lineage, including monocytes, mature macrophages, and granulocytes, all of which are involved in innate immune responses [24]. The myeloid lineage-specific AC7 KO mice were shown to express AC7 mRNA in the bone marrow-derived macrophages and peritoneal macrophages at a level of about 10% of wild-type mice [14]. Innate immune responses serve as the body’s first line of defense against pathogens, offering a rapid and nonspecific reaction to infections. Myeloid cells play a crucial role in these responses. We have shown that knocking out AC7 in a cell line derived from microglial cells, the brain’s resident macrophages, impairs immune functions and diminishes cells’ ability to respond to ethanol [25].

In this study, we used the myeloid lineage-specific AC7 KO mice to explore the effects of AC7 deletion in myeloid cells on LPS-induced systemic immune responses. We examined the effects of ethanol, acute and chronic, on LPS-induced mortality and the expression of selected cytokine mRNAs in the lung and liver. We observed significant effects of AC7 KO and ethanol on animals’ immune responses. In some cases, we observed significant interactions between the effects of AC7 KO and ethanol. The results suggest that AC7 plays a role in ethanol’s effects on animals’ immune responses.

## 2. Results

### 2.1. Effects of Acute Ethanol Treatment on Lipopolysaccharide (LPS)-Induced Mortality in WT and Myeloid-Specific AC7 KO Mice

**Figure 1:**
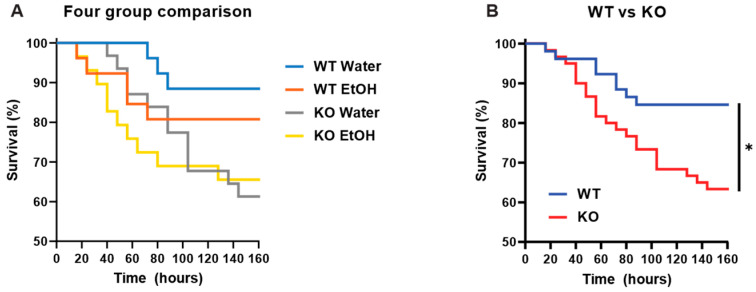
Effects of acute ethanol treatment on LPS-induced mortality in WT and myeloid-specific AC7 KO male mice. (**A**) LPS-challenged WT and LPS-challenged myeloid-specific AC7 KO were gavaged with water or ethanol. Ten mice per group were used in one experiment, which was repeated three times. Thus, a total of 30 animals per group was used. The survival rate was observed for 160 h post-LPS challenge. (**B**) Data for water and ethanol are combined and plotted for genotypes. The differences were analyzed using the Kaplan–Meier Survival Analysis (Logrank test). * indicates *p* < 0.05.

To estimate the effects of acute ethanol and myeloid-specific AC7 KO on LPS-induced mortality, water- or alcohol-treated WT and AC7-knockout male mice were challenged with LPS (LD50 dose: 9.3 mg/kg), and mortality was recorded over the period of 160 h, post-LPS challenge. LD50 of LPS in wild-type (WT) male mice was estimated as 9.3 mg/kg by i.p. injection of different doses of LPS to monitor the survival rates (see Supplementary Figure S1). While acute ethanol treatment slightly suppressed the survival rate of LPS-challenged WT male mice, the LPS-challenged AC7 KO male mice had similar survival rates in water and alcohol treatment groups. Regardless, the alcohol treatment did not significantly affect the LPS-induced mortality in both genotypic groups (Figure 1A). When the results shown in Figure 1A were plotted for genotype difference (Figure 1B), 85% of WT male mice survived LPS-induced systemic inflammation, significantly higher than the survival rate of AC7 KO male mice (63%) (Figure 1B).

### 2.2. Effects of Chronic Ethanol Treatment on LPS-Induced Mortality in WT and Myeloid-Specific AC7 KO Mice

**Figure 2:**
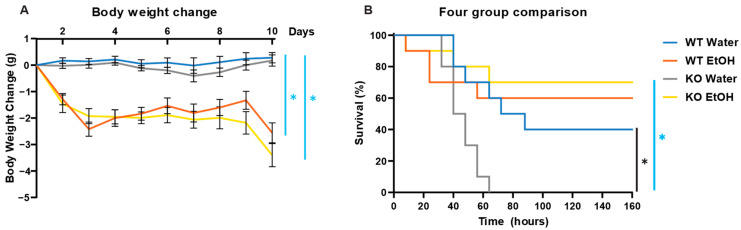
Effects of chronic ethanol treatment on LPS-induced mortality in WT and myeloid-specific AC7 KO male mice. (**A**) Change in body weight of male mice during 10-day gavage. Ten mice per group were used. Blue bars and blue * indicate significant body weight differences between groups (WT Water vs. WT EtOH and KO Water vs. KO EtOH) at day 10 (*p* < 0.05). (**B**) Mice survival rate after chronic ethanol gavage and LPS injection. The differences were analyzed using the Kaplan–Meier Survival Analysis (Logrank test). Black bar and black * indicate a significant difference in survival between water-treated groups (WT vs. AC7 KO), *p* < 0.05. Blue bar and blue * indicate a significant difference in survival within the AC7 KO mice (water vs. ethanol-treated), *p* < 0.05.

To estimate the effects of chronic ethanol treatment (10-day gavage) on male animals, we measured mice’s body weights before gavage every day. Water gavage did not significantly affect the body weight of male mice in both WT and AC7 KO mice. However, ethanol treatment reduced the body weight of WT and AC7 KO male mice until day 3, after which the weights were stabilized until day 10 (Figure 2A). Compared to the WT male mice, myeloid-specific AC7 deficiency had no significant effect on the body weight changes during the 10-day gavage treatment.

To determine the effect of chronic ethanol treatment on the LPS-induced mortality and to determine the role of myeloid cell-specific AC7 in these responses, chronic ethanol-treated WT and chronic ethanol-treated myeloid-specific AC7 KO male mice were challenged with LPS (LD50 dose: 9.3 mg/kg) and mortality was recorded over the period of 160 h, post-LPS-challenge (Figure 2B). The survival rate of water-treated LPS-challenged WT male mice was ~40%. Chronic ethanol treatment slightly increased the WT male mice survival rate to 60%. However, this effect was not statistically significant. No water-treated LPS-challenged AC7 KO male mice survived the LPS challenge, as indicated by a significant difference from water-treated LPS-challenged WT male mice. Compared with water-treated LPS-challenged AC7 KO male mice, the survival rate of ethanol-treated LPS-challenged AC7 KO male mice was improved to ~70%. These data indicate that myeloid cell-specific AC7 deficiency significantly compromises the survivability of LPS-challenged mice. Further, prior chronic ethanol treatment confers protection against LPS-induced mortality in myeloid cell-specific AC7 KO mice (Figure 2B).

### 2.3. Effects of Acute Ethanol Treatment and Myeloid-Specific AC7 KO on Cytokine Expression in the Lung Induced by i.p. Injection of LPS

**Figure 3:**
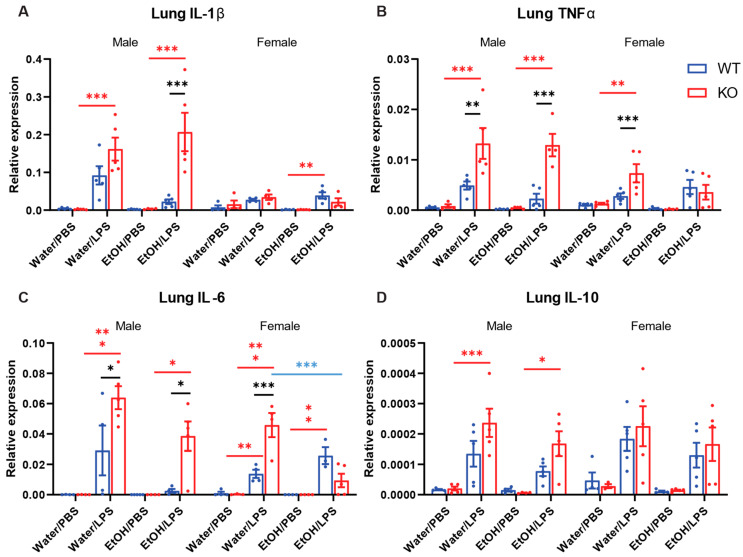
Effects of acute ethanol treatment and myeloid-specific AC7 KO on cytokine expression in the lung induced by i.p. injection of LPS. Mice (n = 3–5) received one oral gavage of either water or 25% (*w*/*v*) ethanol solution (EtOH) followed by i.p. injection of either Dulbecco’s Phosphate Buffered Saline (PBS) or 3 mg/kg LPS, as indicated. The lung tissues were harvested 2 h after the LPS injection. The expression levels of selected cytokine mRNAs were examined by Real-time quantitative PCR (RT-qPCR) (**A**: IL-1β, **B**: TNFα, **C**: IL-6, and **D**: IL-10). The results, including male and female data, were analyzed by 4-way ANOVA. Male and female data were analyzed by 3-way ANOVA for pair-wise comparison, respectively. Differences in pair-wise comparisons are indicated by bars and * (*: *p* < 0.05, **: *p* < 0.01, and ***: *p* < 0.001). The colors indicate compared factors (red: PBS vs. LPS, black: WT vs. AC7 KO, and blue: water vs. ethanol).

To determine the effects of LPS, AC7 KO, and acute ethanol on systemic inflammation, mice were treated with a single ethanol gavage (5 g/kg) and i.p. injection of a sublethal dose of LPS (3 mg/kg), as described in Materials and Methods. Mice were exposed to LPS for two hours. Lung tissue was harvested and used to access cytokine mRNA expression using Real-time quantitative PCR (RT-qPCR). The expression of pro-inflammatory cytokines (IL-1β, TNFα, and IL-6) and an anti-inflammatory cytokine (IL-10) in the lung tissue were measured (Figure 3). The results, including those of males and females, were analyzed using four-way ANOVA (Table 1). Then, male and female data were separately analyzed by three-way ANOVA with Tukey pair-wise comparison for each sex (Table 2 and Table 3, Figure 3). As expected, LPS injection had highly significant effects on all four cytokine expressions. In addition, the impact of genotype was significant for all four cytokine expressions (Table 1). This is mainly derived from the male data (Table 2). Based on the ANOVA results, it appeared that male AC7 KO mice showed higher expression of all four cytokines than male WT mice. In female mice, ANOVA indicated that genotype alone did not significantly affect cytokine expression (Table 3). However, some pair-wise comparisons (TNFα and IL-6) showed higher expression in female AC7 KO mice than in female WT mice when treated with water and LPS (Figure 3B,C). The sex of animals was significant for IL-1β and TNFα expression (Table 1). Male mice appeared to have higher expression of IL-1β and TNFα than female mice (Figure 3A,B). The effect of gavage was significant for IL-6 expression in male mice (Table 2) and significant for IL-6 and IL-10 expressions in female mice (Table 3). Acute ethanol treatment appeared to reduce cytokine expression. Significant interactions existed between genotype and gavage for the expression of TNFα and IL-6 in female mice (Table 3). Ethanol appeared to reduce the expression of TNFα and IL-6 only in AC7 KO female mice (Figure 3B,C).

### 2.4. Effects of Chronic Ethanol Treatment and Myeloid-Specific AC7 KO on Cytokine Expression in the Lung Induced by i.p. Injection of LPS

**Figure 4:**
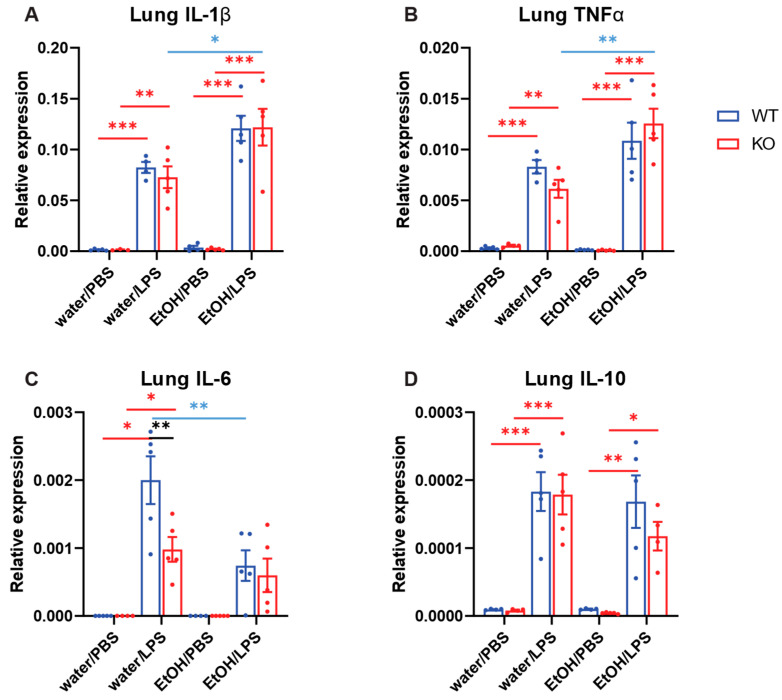
Effects of chronic ethanol treatment and myeloid-specific AC7 KO on cytokine expression in the lung of male mice induced by i.p. injection of LPS. Male mice (n = 3–5) received oral gavage of either water or 25% (*w*/*v*) ethanol solution (EtOH) for 10 days, followed by i.p. injection of either PBS or 3 mg/kg LPS as indicated. The lung tissues were harvested two hours after the LPS injection. The expression levels of selected cytokine mRNAs were examined by RT qPCR (**A**: IL-1β, **B**: TNFα, **C**: IL-6, and **D**: IL-10). The results were analyzed using 3-way ANOVA. Asterisks indicate that differences between the groups are significant (*: *p* < 0.05, **: *p* < 0.01, ***: *p* < 0.001). Red bars and asterisks indicate significant effects of LPS. Blue bars and asterisks indicate the significant effects of chronic ethanol treatment. Black bars and asterisks indicate significant effects of AC7 KO.

In order to determine the effects of chronic ethanol and AC7 deficiency on LPS-induced systemic inflammation, we accessed the cytokine mRNA expression in the lungs of male mice after 10 days of ethanol gavage and i.p. injection of LPS, as described in Materials and Methods. The expression levels of four cytokines (IL-1β, TNFα, IL-6, and IL-10) were examined using RT-qPCR. The results were analyzed by three-way ANOVA followed by Tukey pair-wise comparison (Figure 4 and Table 4). For this experiment, we only focused on male mice. Analyses by three-way ANOVA indicated that LPS had a highly significant effect on all four cytokine expressions, that genotype had a significant effect on only IL-6 expression, and that chronic ethanol had a significant effect on the expression of IL-1β, TNFα, and IL-6, but not on the expression of IL-10. While chronic ethanol treatment appeared to increase the expression of IL-1β and TNFα compared to water treatment, chronic ethanol appeared to decrease IL-6 expression. IL-10 expression is only significantly affected by LPS. Although the ANOVA did not detect any significant interactions between genotype and gavage for the all four cytokine expressions, pair-wise comparisons appeared to show that chronic ethanol treatment decreased IL-6 expression only in WT mice (Figure 4C).

### 2.5. Effects of Acute Ethanol Treatment and Myeloid-Specific AC7 KO on Cytokine Expression in the Liver Induced by i.p. Injection of LPS

**Figure 5:**
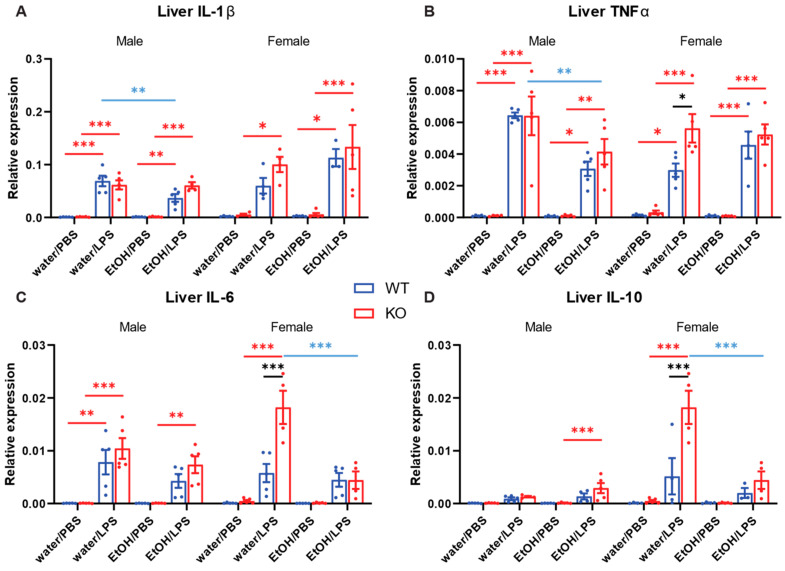
Effects of acute ethanol treatment and myeloid-specific AC7 KO on cytokine expression in the liver induced by i.p. injection of LPS. Mice (n = 3–5) received one oral gavage of either water or 25% (*w*/*v*) ethanol solution (EtOH) followed by i.p. injection of either PBS or 3 mg/kg LPS, as indicated. The expression levels of selected cytokine mRNAs were examined by RT qPCR (**A**: IL-1β, **B**: TNFα, **C**: IL-6, and **D**: IL-10). The results, including male and female data, were analyzed by 4-way ANOVA. Male and female data were analyzed by 3-way ANOVA for pair-wise comparison (Tukey), respectively. Differences in pair-wise comparisons are indicated by bars and * (*: *p* < 0.05, **: *p* < 0.01, and ***: *p* < 0.001). The colors indicate compared factors (red: PBS vs. LPS, black: WT vs. AC7 KO, and blue: water vs. ethanol).

The liver was harvested from the mice described in Section 2.3, and the expression of IL-1β, TNFα, IL-6, and IL-10 mRNA was measured (Figure 5). The results, including those of males and females, were analyzed using four-way ANOVA (Table 5). Then, male and female data were separately analyzed by three-way ANOVA with Tukey pair-wise comparison for each sex (Table 6 and Table 7 and Figure 5). Similar to the expression in the lung, LPS injection significantly induced the expression of all four cytokines. The sex of the mice exhibited significant differences in the expression of IL-1β and IL-10. The genotype and the gavage showed significant effects on TNF, IL-6, and IL-10 expression (Table 5). However, the statistical differences appeared to be derived largely from the data obtained from female mice (Table 6 and Table 7). Significant interactions existed between genotype and gavage for the expression of IL-6 and IL-10, particularly in female mice. The pair-wise comparisons indicated that ethanol significantly reduced the expression of IL-6 and IL-10 in only female AC7 KO mice (Figure 5C,D).

### 2.6. Effects of Chronic Ethanol Treatment and Myeloid-Specific AC7 KO on Cytokine Expression in the Liver Induced by i.p. Injection of LPS

**Figure 6:**
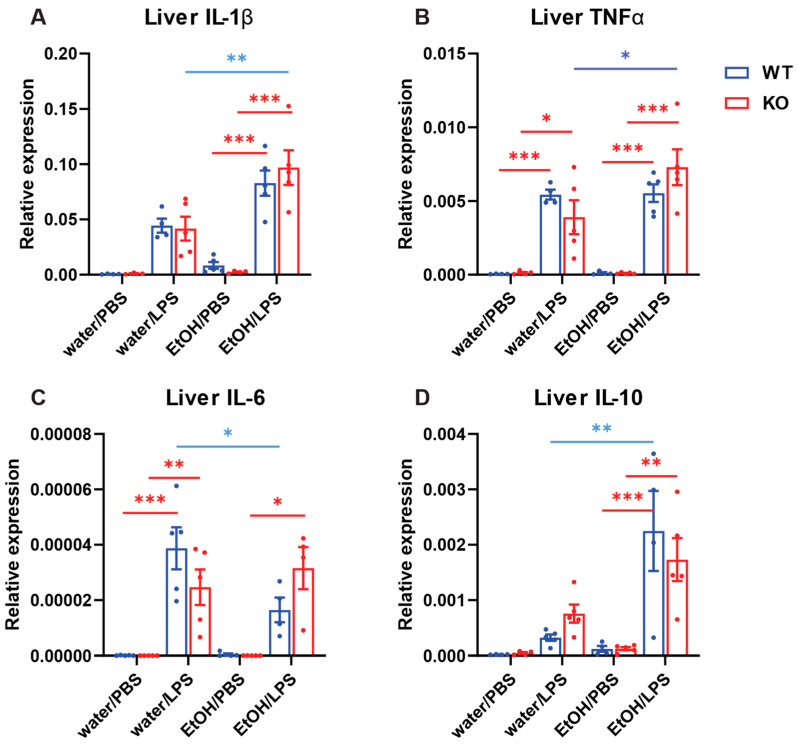
Effects of chronic ethanol treatment and myeloid-specific AC7 KO on cytokine expression in the liver induced by i.p. injection of LPS. Male mice (n = 4–5) received oral gavage of either water or 25% (*w*/*v*) ethanol solution (EtOH) for 10 days, followed by i.p. injection of either PBS or 3 mg/kg LPS as indicated. The expression levels of selected cytokine mRNAs were examined by RT qPCR (**A**: IL-1β, **B**: TNFα, **C**: IL-6, and **D**: IL-10). The results were analyzed using a 3-way ANOVA followed by Tukey pair-wise comparisons. Asterisks indicate that differences between the groups are significant (*: *p* < 0.05, **: *p* < 0.01, ***: *p* < 0.001). Red bars and asterisks indicate significant effects of LPS. Blue bars and asterisks indicate the significant effects of chronic ethanol treatment.

The liver was harvested from the male mice described in Section 2.4, and the expression of IL-1β, TNFα, IL-6, and IL-10 mRNA was measured (Figure 6). The results were analyzed using three-way ANOVA (Table 8). As expected, LPS increased the expression of all four cytokines significantly. Overall, genotype did not significantly affect the expression of all four cytokines, and the gavage significantly affected the expression of IL-1β and IL-10. There was a significant interaction between genotype and gavage for IL-6 expression (Table 8). It appeared that chronic ethanol treatment significantly decreased IL-6 expression only in WT mice (Figure 6C). The ANOVA results suggested that chronic ethanol appeared to increase the expression of IL-1β and IL-10 regardless of genotype (Figure 6A,D).

## 3. Discussion

The current study aims to assess the hypothesis that AC7 is central in regulating alcohol’s effects on immune responses. We employed myeloid lineage-specific AC7 KO mice and examined the effects of ethanol (acute and chronic) on LPS (systemic)-induced immune responses. If the hypothesis is correct, there must be differences in alcohol’s effects on immune responses between WT and AC7 KO mice.

This study employed the myeloid lineage-specific AC7 KO mice as a model [14]. Myeloid cells are a collection of monocytes, macrophages, neutrophils, basophils, erythrocytes, megakaryocytes, and platelets differentiated from common progenitors derived from hematopoietic stem cells [26]. In our animal model, AC7 was knocked out using LysMcre [24], which is mainly expressed in granulocytes and monocytes/macrophages. Previous studies have indicated that AC7 is the dominant isoform of AC in several types of immune cells and plays a vital role in regulating signaling transduction during infection and inflammation [14,21]. These conditional AC7 KO mice appeared healthy and showed no health-related problems under normal laboratory conditions. We bred over 2000 mice in our mouse colony. The overall ratio of mice genotype WT:AC7 KO was very close to the expected ratio of 1:1. We did not observe any viability differences between WT and AC7 KO litter mates. In addition, the sex ratio was also close to 1:1. This is quite a contrast compared to whole-body AC7 KO mice, which exhibit a high embryonic lethal phenotype [21,23].

Intraperitoneal (i.p.) injection of LPS induces innate immune responses in mice. This is a widely used animal model of sepsis [27,28]. In almost all measurements that we carried out in this report, LPS caused an increase in cytokine expression in the lung and liver compared to the control (i.e., administration of Dulbecco’s Phosphate Buffered Saline (PBS)). Compared to PBS injection, LPS effects on cytokine expression were highly significant (Figure 3, Figure 4, Figure 5 and Figure 6 and Table 1, Table 2, Table 3, Table 4, Table 5, Table 6, Table 7 and Table 8). A similar observation was also made for the brain (Figure S2 and Table S1). The results strongly indicate that systemic inflammation was achieved by i.p. injection of LPS as expected.

Alcohol via oral gavage was used as acute and chronic treatment with alcohol in this research. The dose (5.0 g/kg) selected in this study was shown to achieve a blood ethanol concentration of ~67 mM, which is not rare in humans [29] and is well-tolerated by mice [30].

We found that AC7 deficiency significantly increased the mortality of LPS-challenged male mice (Figure 1 and Figure 2B). These results indicate that AC7 expression in myeloid cells enhances animal survival during LPS-induced systemic inflammation. The results are consistent with a previous report that showed mice harboring an AC7-deficient immune system were more sensitive to endotoxic shock and showed a higher mortality [21]. While acute alcohol treatment did not show a significant effect on survival, chronic ethanol treatment significantly improved the survival of LPS-challenged AC7 KO mice but not WT mice (Figure 2B). The results support our hypothesis that AC7 is central in regulating alcohol’s effects on immune responses. Chronic ethanol treatment appeared to compensate for the lack of AC7 expression in AC7 KO myeloid cells. Chronic ethanol treatment appeared to stimulate or induce other signaling pathways that potentially substituted for cAMP signaling in myeloid cells, resulting in increased survival. Alternatively, expression of other isoforms of AC in myeloid cells might increase during chronic alcohol treatment and, thus, compensate for the AC7 deficiency. Many publications have shown that ethanol treatment (acute and chronic) reduces the survival of animals during systemic inflammation caused by bacterial infection [31,32,33,34,35]. We found two publications that show that ethanol increased the survival of mice under LPS-induced inflammation [36,37].

This study used male mice for survival studies and cytokine measurements. For cytokine measurements with acute alcohol treatment, female mice were also included. We observed that female mice showed a different cytokine expression pattern from male mice in the lung and liver (Figure 3 and Figure 5, Table 1, Table 2, Table 3, Table 5, Table 6 and Table 7). In the lung, the expression of IL-1β and TNFα was lower in female mice than in male mice (Figure 3A,B). IL-1β and IL-10 expression in the liver was higher in female mice (Figure 5A,D). Differences in the cytokine expressions between male and female mice are expected. Earlier research has reported that due to the effects of female sex hormones, female rats subjected to sepsis showed less liver and lung tissue damage and less systemic endotoxemia than male rats [38]. An epidemiologic study in humans has also indicated that the prevalence of severe sepsis is lower in women than in men [39]. Female sex hormones exhibit protective effects that contribute to the natural advantages of women under septic conditions [40]. Significantly elevated levels of pro-inflammatory cytokines, including TNFα, IL-1, and IL-6, have been observed in endotoxemia/sepsis in males compared to females. Conversely, anti-inflammatory mediators were increased in females [40,41,42]. One previous study suggests that higher LPS-induced TNFα secretion by monocytes in males is correlated to a sex-specific decrease in AC expression in male monocytes [43].

To assess our hypothesis that AC7 is a central player in regulating alcohol’s effects on immune responses, we examined the effects of ethanol and mouse genotype (WT or AC7 KO) on the expression of cytokines. We are particularly interested in the statistical significance of the interaction between genotype and gavage using ANOVA. The significance of this interaction suggests that AC7 plays a role in the observed effects of alcohol on cytokine expression. Acute ethanol decreased the lung expression of TNFα and IL-6 in female AC7 KO mice but not in female WT mice (Figure 3B,C and Table 3). Similarly, acute ethanol decreased the liver expression of IL-6 and IL-10 in female AC7 KO mice but not in female WT mice (Figure 5C,D, Table 7). Chronic ethanol decreased the liver expression of IL-6 in male AC7 WT mice but not in male AC7 KO mice (Figure 6C and Table 8). In addition, acute alcohol treatment reduced MIP-1β accumulation in the peritoneal cavity only in WT male mice (Figure S3 and Table S2). The results suggest that in some measurements, ethanol’s effects on cytokine expression are affected by AC7 expression in myeloid lineage cells.

The regulation of the expression of cytokines (IL-1β, TNFα, IL-6, and IL-10) by AC7 does not seem robust. This may contribute to the observation that there are only a few cases in which AC7 KO affects cytokine expression. For male mice shown in Figure 3, genotype had a significant effect on all four cytokine expressions in the lung (Table 2). In contrast, no significant genotype effects were seen in the liver (Figure 5 and Table 6). For male mice shown in Figure 4 and Figure 6, a significant effect of genotype was only observed for IL-6 expression in the lung (Table 4). It appears that the AC7 effect on cytokine expression is organ-specific and that chronic ethanol treatment eliminates the AC7 effect on cytokine expression (except on IL-6). Earlier research indicated that LPS attenuates mRNA levels of AC7 in different tissues [44]. AC7 mRNA levels in the lung are not significantly suppressed by LPS treatment. However, LPS treatment significantly suppresses AC7 mRNA levels in the liver. For long-term LPS treatment of animals, we speculate that suppressed AC7 expression in the liver by LPS could contribute to cytokine expression differences between the lung and liver.

We expected that the AC7 KO would increase the expression of pro-inflammatory cytokines, IL-1β and TNFα, while reducing anti-inflammatory IL-10 expression [11]. Regarding IL-6, cyclic AMP was shown to increase its expression in monocytes [45]; thus, we expected that AC7 KO would decrease its expression. However, we observed in several cases that AC7 KO has no effect, or the opposite effect, on cytokine expression. These data suggest that AC7’s role in the expression of cytokines in vivo is more complicated than in vitro cell culture and that other factors are involved in the regulation. Based on the human single-cell RNA expression data [46], we expect that the expression of IL-1β and IL-10 is limited to macrophages in the lung and Kupffer cells (resident macrophages) in the liver. The expression of TNFα is also seen in lymphocytes (T and B cells) in these organs. On the other hand, the expression of IL-6 is more widespread in the lung and liver. Especially in the lung, the major source of IL-6 expression could be endothelial cells [46]. The AC7 KO mice used in this study have LysMcre-mediated gene knockout, and AC7 knockout is limited to the cells of the myeloid lineage. Thus, it is interesting to see a robust AC7 KO effect on IL-6 expression (Figure 3C, Figure 4C and Figure 5C). The human single-cell RNA expression data [46] indicate that macrophages in the lung and Kupper cells in the liver express AC7 predominantly among the 10 different isoforms. Similarly, both rat alveolar macrophages and Kupper cells express AC7 predominantly [44]. Thus, we expect the AC7 KO mice in this study to express very small activity of adenylyl cyclase. Our recent study showed that this is the case in the AC7 knockout microglia cell line BV2 [25]. However, we cannot rule out the compensatory expression of other AC isoforms in myeloid lineage cells, including macrophages in the AC7 KO mice, which may influence cytokine expression in AC7 KO mice.

Many reports are showing the opposite effect of ethanol on cytokine expression. Ethanol acutely suppresses pro-inflammatory cytokine expression, while chronic ethanol enhances the expression [1,3,47]. However, there are also reports that show different effects of ethanol on cytokine expression [30,48]. Effects of ethanol treatment on cytokine expression can differ depending on the duration of ethanol treatment, the dose of ethanol, the cause of inflammation, the tissues examined, and the time of tissue sampling. We observed that acute ethanol significantly decreased the expression of IL-1β and TNFα in the liver of WT male mice (Figure 5A,B). Chronic ethanol decreased IL-6 expression in the lung and liver of WT male mice (Figure 4C and Figure 6C) and increased IL-10 expression in the liver of WT male mice (Figure 6D).

In summary, we showed in this study that myeloid lineage-specific AC7 KO significantly changed the inflammatory responses of mice. In some measurements, ethanol’s effect on inflammatory response was modified by the AC7 KO. The results indicate that AC7 plays a significant role in ethanol’s effects on systemic inflammation in mice. However, the effects of AC7 KO and ethanol were not as robust as we expected. In addition, the effects of AC7 KO on the expression of certain cytokine mRNA were not consistent depending on the sex, the tissue, and the alcohol treatment. Due to these limitations, we feel it is difficult to draw definitive conclusions from this study regarding the role of AC7 and ethanol in the immune response of animals. This is the first study to explore the effects of AC7 KO combined with ethanol treatment on the innate immune system using an in vivo model. The observations presented in this study warrant further investigation of the underlying mechanisms by which AC7 and ethanol influence the innate immune system.

It would be interesting to examine how myeloid lineage-specific AC7 KO affects ethanol effects on sepsis caused by infection of pathogenic bacteria or to examine the effects of AC7 KO in other cell types involved in an inflammatory response.

## 4. Materials and Methods

### 4.1. Animal Handling

Myeloid lineage-specific AC7 KO transgenic mice were obtained from UT Southwestern Medical Center [14]. AC7^fl/fl^, LysM^+/+^ mice and AC7^fl/fl^, LysM^cre/+^ mice were bred to generate WT (AC7^fl/fl^, LysM^+/+^) and AC7 KO (AC7^fl/fl^, LysM^cre/+^) mice. The mouse genotypes were identified by PCR of genomic DNA isolated from tail biopsy when mice reached 3 to 3.5 weeks old. All experimental procedures involving animals in this study were reviewed and approved by the Institutional Animal Care and Use Committee at Louisiana State University. Male and female mice, 8–12 weeks old and weighing 20–25 g, were used in this study. Mice were housed at the animal facility following LSU Division of Laboratory Animal Medicine guidelines.

### 4.2. Animal Survival Study

To estimate the LD50 of LPS for mice, WT male mice were divided into 5 groups (n = 5) randomly. They were given LPS at 5 different doses (5 mg/kg, 7 mg/kg, 10 mg/kg, 15 mg/kg, and 30 mg/kg) by intraperitoneal (i.p.) injection. Mice were monitored every 8 h for up to 7 days and assessed for mortality induced by LPS (Supplementary Figure S1A). LD50 was calculated by three-parameter logistic regression using the calculator offered by ATT Bioquest (https://www.aatbio.com/tools/ld50-calculator (accessed on 28 August 2020)). The LD50 dose of LPS for male mice was determined as 9.3 mg/kg (Supplementary Figure S1B).

For the survival study with acute alcohol treatment, 20 WT and 20 AC7 KO male mice were divided into 4 groups (n = 10 for each group) (Table 9). Mice in groups WT Water and KO Water received sterile water (mouse weight (g) × 20 μL = μL water) by gavage, while mice in groups WT-EtOH and KO-EtOH received 25% (*w*/*v*) ethanol solution in water to achieve 5 g/kg ethanol by gavage. At 30 min post-gavage, all mice were challenged with LPS (LD50: 9.3 mg/kg) through the intra-peritoneal (i.p) route. Mice were observed every 8 h for up to 7 days and assessed for clinical signs and mortality induced by LPS.

A chronic alcohol-treated mouse model was established by administering 25% (*w*/*v*) ethanol solution in water to achieve 5 g/kg ethanol for 10 days via daily single oral gavage. For the survival study, 20 WT and 20 AC7 KO male mice were divided into 4 groups (n = 10 for each group) (Table 9): mice in groups WT-Water and KO-Water received sterile water for 10 days (by gavage) and challenged with an i.p. injection of LPS (LD50: 9.3 mg/kg) 30 min after Day 10 water gavage, while mice in groups WT-EtOH and KO-EtOH received 5 g/kg ethanol for 10 days (by gavage) and challenged with an i.p. injection of LPS (LD50: 9.3 mg/kg), 30 min after Day 10 ethanol gavage. Mice were monitored every 8 h for up to 7 days and assessed for clinical signs and mortality induced by LPS.

### 4.3. Ethanol Effects on Intra-Peritoneal LPS-Challenged Mice Model

The acute alcohol-treated mice model was established by administering 25% (*w*/*v*) ethanol in water to achieve 5 g/kg ethanol via single oral gavage. The chronic alcohol-treated mice model was established by administering 25% (*w*/*v*) ethanol in water to achieve 5 g/kg ethanol for 10 days via daily single oral gavage. For both acute and chronic models, LPS was administered at a sub-lethal dose (3 mg/kg) via i.p. injection 30 min after the final ethanol gavage. Whole experimental groups are shown in Table 10. Typically, 4–5 mice per group were used. At 2 h post-LPS challenge, mice were euthanized by CO_2_. Peritoneal lavage was performed by instilling 2 mL of sterile Dulbecco’s Phosphate Buffered Saline (PBS) into the abdominal cavity and retrieving about 1.5 mL of lavage fluid. The peritoneal lavage fluid (PLF) was harvested for cytokine measurement. Approximately 50 mg of liver, lung, and brain tissues were harvested for cytokine mRNA expression analysis.

### 4.4. RNA Extraction, Reverse Transcription, and Quantitative PCR

Total RNA was extracted using TRI reagent (TR 118, Molecular Research Center, Cincinnati, OH, USA) [49] and dissolved in DNase- and RNase-free water. cDNA was synthesized using the High-Capacity cDNA Reverse Transcription Kit (Applied Biosystems, Foster City, CA, USA). Real-time quantitative PCR (RT-qPCR) was performed by a SYBR green-based detection system using an Applied Biosystems 7300 Real-Time PCR System and 7300 System software (Applied Biosystems, Foster City, CA, USA). Cytokine (IL-1β, IL-6, IL-10, and TNF-α) mRNA expression was measured by RT-qPCR by the ΔCT method using β-actin mRNA as an internal control [50]. Primers were designed to span exon–exon junctions using Primer-BLAST (https://www.ncbi.nlm.nih.gov/tools/primer-blast/ (accessed on 20 October 2018)) [51]. The primer sequences were examined using Blast (https://blast.ncbi.nlm.nih.gov/Blast.cgi?PROGRAM=blastn&PAGE_TYPE=BlastSearch&LINK_LOC=blasthome (accessed on 20 October 2018)) and Primer-Blast to ensure they were unique for the target gene. We designed them so that their melting temperatures are 60 ± 1 °C, and GC% is around 50%. We tested the primers by RT-qPCR followed by melting curve analysis and confirmed that they generated PCR products with one unique melting peak. All the primers are listed in Table 11. Primers were produced by Integrated DNA Technologies (Coralville, IA, USA).

### 4.5. Assessment of Cytokines in Peritoneal Lavage Fluid 

Samples were centrifuged at 2000× *g* for 5 min. The supernatant was used for cytokine assessment, and the cell pellet was used for cell counting. Mouse cytokine levels were assayed in cell-free PLF using a Luminex-XMAP-based assay (MCYTOMAG-70K, EMD Millipore, St. Louis, MO, USA), according to the manufacturer’s instruction. This reagent should detect G-CSF, GM-CSF, IFN-γ, IL-1α, IL-1β, IL-2, IL-4, IL-5, IL-6, IL-7, IL-9, IL-10, IL-12 (p40), IL-12 (p70), IL-13, IL-15, IL-17, IP-10, KC, MCP-1, MIP-1α, MIP-1β, MIP-2, RANTES, and TNF-α.

### 4.6. Statistical Analysis

Survival curves were compared using Kaplan–Meier Survival Analysis (Logrank test) using SigmaPlot version 12.5 (SyStat Software, San Jose, CA, USA). Other data are represented as mean ± standard error of the mean (SEM). Three-way ANOVA and four-way ANOVA followed by Tukey pair-wise comparisons were used to analyze the statistical significance of differences among groups, as appropriate, using JMP Pro version 18 (JMP Statistical Discovery, Cary, NC, USA). A statistically significant difference was defined as *p* < 0.05.

## Figures and Tables

**Table 1:** *p*-values for factors affecting mRNA expression shown in Figure 3 detected by 4-way ANOVA. The values shown in red are significant.

	LPS	Sex	Genotype	Gavage	Genotype X Gavage
IL-1β	<0.0001	<0.0001	0.001	0.5389	0.2382
TNFα	<0.0001	0.0059	<0.0001	0.1439	0.4643
IL-6	<0.0001	0.1281	0.0011	0.0028	0.0670
IL-10	<0.0001	0.0893	0.0195	0.0088	0.3088

**Table 2:** *p*-values for factors affecting mRNA expression in male mice shown in Figure 3 detected by 3-way ANOVA. The values shown in red are significant.

	LPS	Genotype	Gavage	Genotype X Gavage
IL-1β	<0.0001	0.0004	0.7104	0.0736
TNFα	<0.0001	<0.0001	0.3928	0.5889
IL-6	<0.0001	0.0020	0.0184	0.9521
IL-10	<0.0001	0.0299	0.0938	0.7754

**Table 3:** *p*-values for factors affecting mRNA expression in female mice shown in Figure 3 detected by 3-way ANOVA. The values shown in red are significant.

	LPS	Genotype	Gavage	Genotype X Gavage
IL-1β	<0.0001	0.8998	0.2772	0.0960
TNFα	<0.0001	0.2179	0.1973	0.0485
IL-6	<0.0001	0.2074	0.0362	0.0004
IL-10	0.0091	0.1352	0.0376	0.3160

**Table 4:** *p*-values for factors affecting mRNA expression in male mice shown in Figure 4 detected by 3-way ANOVA. The values shown in red are significant.

	LPS	Genotype	Gavage	Genotype X Gavage
IL-1β	<0.0001	0.7218	0.0035	0.7540
TNFα	<0.0001	0.9194	0.0041	0.1907
IL-6	<0.0001	0.0432	0.0057	0.1219
IL-10	<0.0001	0.3528	0.2418	0.4514

**Table 5:** *p*-values for factors affecting mRNA expression in Figure 5 detected by 4-way ANOVA. The values shown in red are significant.

	LPS	Sex	Genotype	Gavage	Genotype X Gavage
IL-1β	<0.0001	0.0007	0.1206	0.3080	0.8335
TNFα	<0.0001	0.5211	0.0387	0.0313	0.6356
IL-6	<0.0001	0.5084	0.0007	<0.0001	0.0218
IL-10	<0.0001	<0.0001	0.0003	0.0019	0.0394

**Table 6:** *p*-values for factors affecting mRNA expression in male mice shown in Figure 5 detected by 3-way ANOVA. The values shown in red are significant.

	LPS	Genotype	Gavage	Genotype X Gavage
IL-1β	<0.0001	0.3042	0.0532	0.0659
TNFα	<0.0001	0.5019	0.0012	0.4825
IL-6	<0.0001	0.1291	0.0787	0.8908
IL-10	<0.0001	0.0887	0.0842	0.3108

**Table 7:** *p*-values for factors affecting mRNA expression in female mice shown in Figure 5 detected by 3-way ANOVA. The values shown in red are significant.

	LPS	Genotype	Gavage	Genotype X Gavage
IL-1β	<0.0001	0.2250	0.1154	0.7149
TNFα	<0.0001	0.0236	0.5390	0.1509
IL-6	<0.0001	0.0017	0.0002	0.0017
IL-10	<0.0001	0.0033	0.0016	0.0329

**Table 8:** *p*-values for factors affecting mRNA expression shown in Figure 6 detected by 3-way ANOVA. The values shown in red are significant.

	LPS	Genotype	Gavage	Genotype X Gavage
IL-1β	<0.0001	0.8328	0.0008	0.7041
TNFα	<0.0001	0.8887	0.0949	0.1271
IL-6	<0.0001	0.9688	0.2514	0.0328
IL-10	<0.0001	0.9464	0.0006	0.2323

**Table 9:** Effects of ethanol treatment and myeloid-specific AC7 KO on LPS-induced mortality.

Group	Genotype	Oral Gavage	i.p. Injection
WT Water	WT	Water	LPS
WT EtOH	WT	Ethanol	LPS
KO Water	AC7 KO	Water	LPS
KO EtOH	AC7 KO	Ethanol	LPS

**Table 10:** Experimental groups for effects of ethanol treatment and myeloid-specific AC7 KO on LPS-induced immune responses.

Group	Genotype	Oral Gavage	i.p. Injection
WT Water/PBS	WT	Water	PBS
WT Water/LPS	WT	Water	LPS
WT EtOH/PBS	WT	Ethanol	PBS
WT EtOH/LPS	WT	Ethanol	LPS
KO Water/PBS	AC7 KO	Water	PBS
KO Water/LPS	AC7 KO	Water	LPS
KO EtOH/PBS	AC7 KO	Ethanol	PBS
KO EtOH/LPS	AC7 KO	Ethanol	LPS

**Table 11:** RT-qPCR primers.

	Forward (3′–5′)	Reverse (3′–5′)
IL-1β	GCACTACAGGCTCCGAGATGA	GTCGTTGCTTGGTTCTCCTTGT
IL-6	GAGGATACCACTCCCAACAGACC	CTGCAAGTGCATCATCGTTGTTCA
IL-10	ATAACTGCACCCACTTCCCA	GGGCATCACTTCTACCAGGT
TNF-α	CACCACGCTCTTCTGTCTACTG	TGCTCCTCCACTTGGTGGTT
β-actin	CCTTCTACAATGAGCTGCGTGT	CTGGATGGCTACGTACATGGC

## Data Availability

Data is contained within the article and Supplementary Materials.

## Supplementary Materials

The following supporting information can be downloaded at: https://www.mdpi.com/article/10.3390/ijms252312831/s1.
