# Supplementary material for: Role of Myeloid Cell-Specific Adenylyl Cyclase Type 7 in Lipopolysaccharide- and Alcohol-Induced Immune Responses
Source: Int J Mol Sci. 2024 Nov 28;25(23):12831. doi: 10.3390/ijms252312831 (PMC11641285; doi:10.3390/ijms252312831)
Supplement: Supplementary file 1 [file ijms-25-12831-s001.zip › ijms-3252982-supplementary.pdf]

Supplementary Materials

Role of Adenylyl Cyclase Type 7 Expressed in Myeloid Cells in Immune Response of Mouse against LPS and Drinking Alcohol

Yawen Hu, Sonika Patial, Yogesh Saini, and Masami Yoshimura

Figure S1

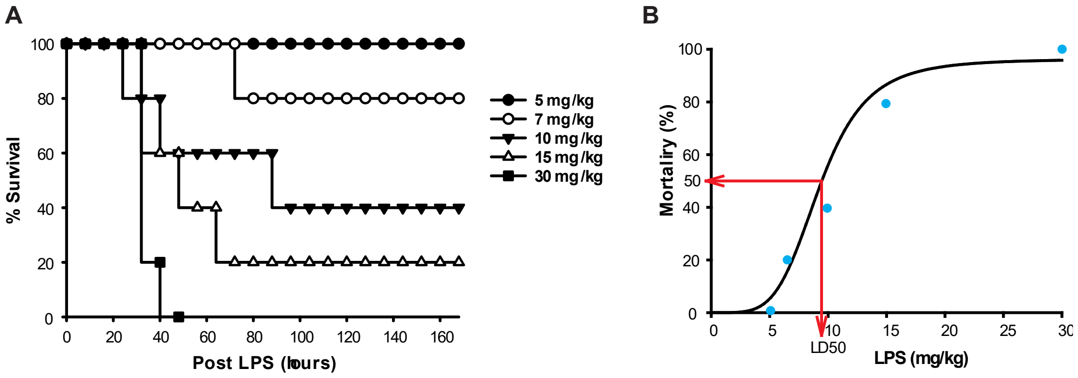

Figure S1. Determination of LD50

LD50 of LPS in WT mice was determined by IP challenging with different doses of LPS to monitor the survival rates. Male mice were challenged with 30 mg/kg, 15 mg/kg, 10 mg/kg, 7 mg/kg, and 5 mg/kg LPS, respectively, and the survival curves were plotted (Figure S1A). The LD50 was estimated at 9.3 mg/kg (Figure S1B).

Figure S2

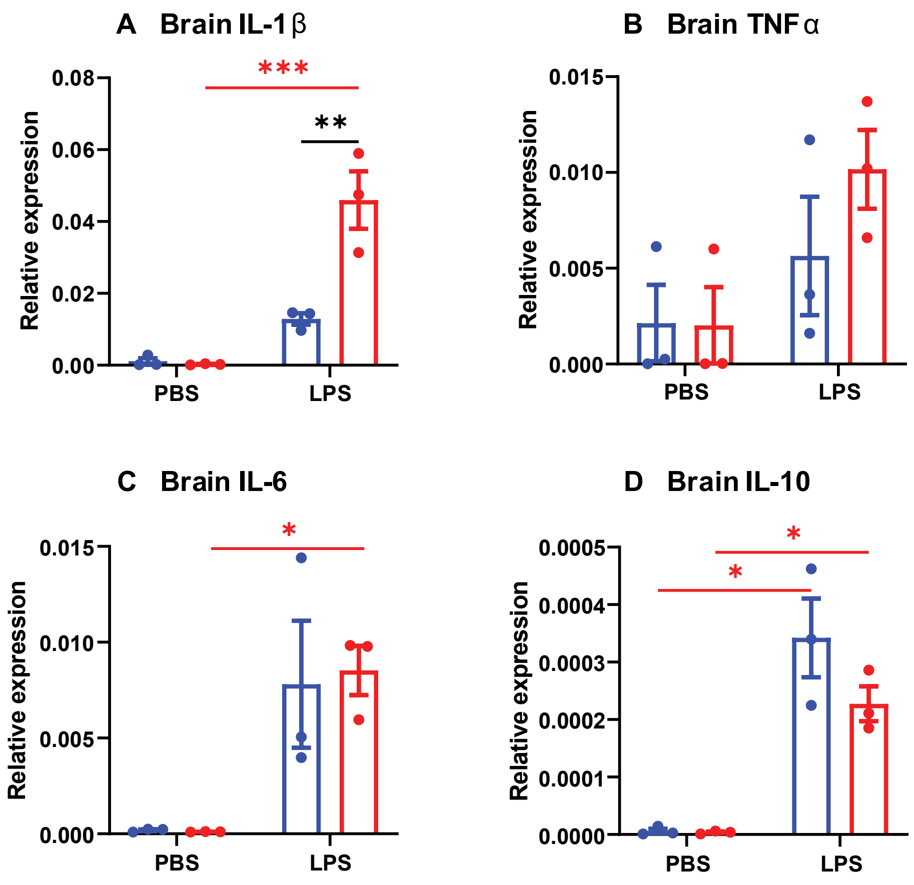

Figure S2. Effects of myeloid-specific AC7 KO on cytokine expression in the brain induced by i.p. injection of LPS.

Male mice (n = 3) received i.p. injection of either PBS or 3 mg/kg LPS as indicated. The brain tissues were harvested two hours after the LPS injection. The expression level of selected cytokine mRNA was examined by RT-qPCR. The results were analyzed by 2-way ANOVA followed by Tukey pair-wise comparison. Asterisks indicate that differences between the groups are significant (*: $p < 0.05$, **: $p < 0.01$, ***: $p < 0.001$). Red bars and asterisks indicate significant effects of LPS. Black bars and asterisks indicate the significant effects of the genotype.

Table S1. p values for factors affecting mRNA expression in Figure S2. The values shown in red are significant.

	LPS	Genotype
IL-1β	<0.0001	0.0044
TNFα	0.0369	0.3721
IL-6	0.0019	0.8630
IL-10	<0.0001	0.1575

The results were analyzed by 2-way ANOVA.

Figure S3

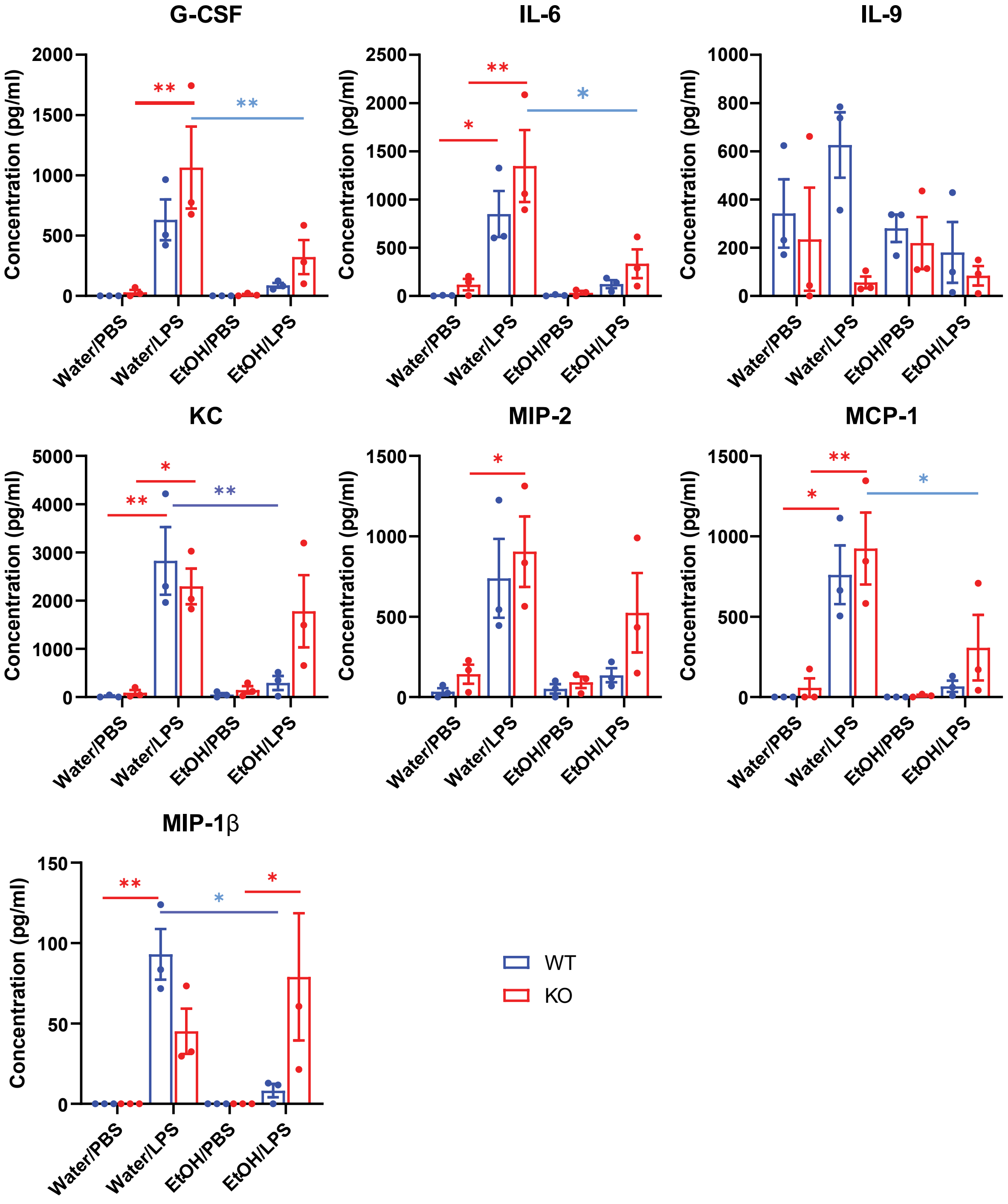

Figure S3. Effects of acute ethanol treatment and myeloid-specific AC7 KO on the accumulation of cytokines and chemokines in peritoneal lavage fluid induced by i.p. injection of LPS.

Mouse cytokine/chemokine levels were examined in peritoneal lavage fluid using a Luminex-XMAP-based assay. Out of 25 cytokines/chemokines examined (G-CSF, GM-CSF, IFN-γ, IL-1α, IL-1β, IL-2, IL-4, IL-5, IL-6, IL-7, IL-9, IL-10, IL-12 (p40), IL-12 (p70), IL-13, IL-15, IL-17, IP-10, KC, MCP-1, MIP-1α, MIP-1β, MIP-2, RANTES, TNF-α), 7 were detected, as shown in panels A to G. The results were analyzed by 3-way ANOVA followed by Tukey pair-wise comparisons. n = 3. Red bars and asterisks indicate significant effects of LPS. Blue bars and * indicate significant effects of acute ethanol treatment. *: $p < 0.05$ and **: $p < 0.01$.

Table S2. p values for factors affecting cytokine/chemokine accumulation in Figure S3. The values shown in red are significant.

	LPS	Genotype	Gavage	Genotype X Gavage
G-CSF	0.0001	0.1008	0.0054	0.5978
IL-6	<0.0001	0.0014	0.0916	0.4412
IL-9	0.7095	0.0268	0.1680	0.1493
KC	<0.0001	0.3222	0.0166	0.0839
MIP-2	0.0002	0.1131	0.0281	0.7191
MCP-1	<0.0001	0.2094	0.0017	0.9407
MIP-1β	0.0001	0.6187	0.2746	0.0181

The results were analyzed by 2-way ANOVA.
